# A Novel Multilayer Composite Membrane for Wound Healing in Mice Skin Defect Model

**DOI:** 10.3390/polym12030573

**Published:** 2020-03-04

**Authors:** Yuyu Qiu, Qingqing Wang, Yajun Chen, Shufang Xia, Wei Huang, Qufu Wei

**Affiliations:** 1 Wuxi School of Medicine, Jiangnan University, Wuxi 214122, China; yuyuqiu1102@aliyun.com (Y.Q.); xiashufang@jiangnan.edu.cn (S.X.); huangwei83721@163.com (W.H.); 2 Key Laboratory of Eco-Textiles, Ministry of Education, Jiangnan University, Wuxi 214122, China; qqwang@jiangnan.edu.cn (Q.W.); m18861824955@163.com (Y.C.)

**Keywords:** multilayer composite membrane, wound dressing, wound healing, antibacterial activity, biocompatibility

## Abstract

To develop a wound dressing material that conforms to the healing process, we prepared a multilayer composite (MC) membrane consisting of an antibacterial layer (ABL), a reinforcement layer (RFL), and a healing promotion layer (HPL). Biocompatible zein/ethyl cellulose (zein/EC) electrospun nanofibrous membranes with in situ loaded antibacterial photosensitizer protoporphyrin (PPIX) and healing promotion material vaccarin (Vac) were, respectively, chosen as the ABL on the surface and the HPL on the bottom, between which nonwoven incorporated bacterial cellulose (BC/PETN) as the HPL was intercalated to enhance the mechanical property. Photodynamic antibacterial activity against *Staphylococcus aureus* and *Pseudomonas aeruginosa* was confirmed by the enlarged inhibition zones; meanwhile, satisfactory biocompatibility of the HPL was verified by scanning electronic microscopy (SEM) of L929 cells cultured on its surface. The potential effects on wound healing in a mice skin defect model of the MC membranes were also evaluated. The animal experiments demonstrated that the wound healing rate in the MC group was significantly increased compared with that in the control group (*p* < 0.05). Histopathological observation revealed an alleviated inflammatory response, accompanied with vascular proliferation in the MC group. The MC membranes significantly promoted wound healing by creating an antibacterial environment and promoting angiogenesis. Taken together, this MC membrane may act as a promising wound dressing for skin wound healing.

## 1. Introduction

An open wound is a type of injury that happens when the skin is punctured, torn, or cut. It specifically refers to the epidermis of the skin damaged by a sharp injury in pathology [1]. The main factors that promote wound healing include mechanical protection of the localized wound, prevention of infection, absorption of wound exudates, and maintenance of the microenvironment [2]. Modern wound dressings are technical products that create an ideal healing environment for wounds [3]. Given these requirements, the ideal wound dressing should be designed with certain specialties, including excellent biocompatibility, good mechanical properties as barriers, the ability to create a native extracellular matrix to stimulate cell migration, ability to remove excessive exudates, and sustained drug release properties to prevent bacterial infections and promote tissue structure proliferation and recombination [4,5]. No particular dressing has all of these characteristics, which is why the dressing is selected only after strict assessment. Therefore, composite films composed of biocompatible polymer and multifunctional drugs as wound dressings have attracted great attention in the field of wound healing [6].

Recently, electrospinning membranes and scaffolds have aroused great interest because of their biomedical applications in wound dressing, such as drug delivery, cell migration scaffolds, and transportation of gas and nutrients [7,8]. Compared with the traditional wound dressing, electrospinning nanofibrous membranes have several advantages, including simulation of a natural extracellular matrix due to three-dimensional (3D) reticulate structure [9,10], small pore size preventing bacteria invasion [11], high porosity to accommodate active ingredients and drug molecular particles [12], and high surface area to release loaded drugs effectively [13]. Thus, electrospun composite membranes have been widely used in the research areas of wound dressings. Since most electrospinning membranes are usually used to load single drug molecules, the composite membranes cannot achieve multiple drug effects simultaneously, which thus makes it difficult to meet clinical treatment needs [14]. Therefore, it is desirable in wound healing research that multiple drugs be loaded via the electrospinning procedure to achieve multi-effects, such as creating an antibacterial environment and promoting cell proliferation.

Winter proposed the concept of a moist healing environment, which creates and sustains the ideal conditions to activate autolytic debridement of the wound, protecting newly formed cells, promoting angiogenesis and epithelial regeneration, reducing pain, and protecting the wound from bacteria and contamination [15,16]. Foam, gel, hydrogel, and aerosol are common types of occlusive wound dressings, which maintain the proper humidity in the wound bed. Bacterial cellulose (BC), synthesized by *Acetobacter xylinum*, consists of nanofibers with a three-dimensional structure, bound together by hydrogen of inter-fibers and intra-fibers to form a dried-state or hydrogel [17]. With the unique properties of high hydrophilicity, tensile strength, crystallinity, and good biocompatibility, it is a promising material used in wound healing applications. Photodynamic antibacterial chemotherapy (PACT) is a method of producing reactive oxygen species to induce antibacterial effects with visible lights combined with photosensitizer molecules. Porphyrin compounds are commonly used photosensitizers because of their highly active oxygen production. Studies showed [18,19] that the combination of protoporphyrin IX (PPIX) and BC membrane has a significant antibacterial effect on *Staphylococcus aureus* and *Escherichia coli.*

**Figure 1:**
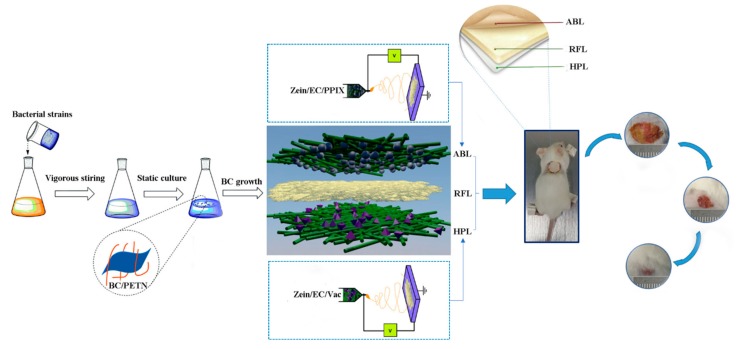
Schematic illustration of the preparation of multilayer composite (MC) membrane by electrospinning. ABL, antibacterial layer; RFL, reinforcement layer; HPL, healing promotion layer; BC, bacterial cellulose.

In this work, PPIX and Vaccarin (Vac) particles were, respectively, impregnated into zein/EC fibers by electrospinning and used as an antibacterial layer (ABL) on the top and healing promotion layer (HPL) on the bottom. PETN was immersed into BC culture solution to develop a reinforcement layer (RFL). The above layers were further compounded to obtain multilayer composite (MC) membranes (Figure 1). The morphology, component, and mechanical properties of the prepared membranes were evaluated by scanning electronic microscopy (SEM), Fourier transform infrared spectroscopy (FTIR), and an electronic universal testing machine, respectively. In addition, the hygroscopicity, antibacterial activity, biocompatibility in vitro, and wound healing in vivo of the as-prepared membranes were also assessed. This study paves an innovated way for creating multi-effective composite membranes, which would have a positive effect in the design of next-generation wound dressings.

## 2. Materials and Methods

### 2.1. Materials

Vaccarin was purchased from Shanghai Shifeng Technology Co., Ltd. (Shanghai, China). Protoporphyrin IX (PPIX, MW = 562.66) was bought from Shanghai Vita Chemical Reagent Co., Ltd (Shanghai, China). Zein (MW = 35,000) was purchased from Sigma-Aldrich (St. Louis, MO, USA) Company. Ethyl cellulose (EC; 6–9 mPa s) was obtained from Aladdin Chemistry Co., Ltd. (Shanghai, China). *Gluconacetobacter xylinus* was cultured by our own lab. Polyester spun-laced nonwovens (40 g/m^2^) were provided by Jiangyin Haiyue Non-woven Fabric Co., Ltd (Wuxi, China). The mouse skin fibroblast cells (L929 cells) were obtained from Shanghai Institutes for Biological Sciences. ICR male mice were gained from Shanghai Slac Laboratory Animal Co., Ltd. (Shanghai, China). All of the chemicals were of analytical grade. All animal experiments were performed in accordance with the Guidelines for the Care and Use of Laboratory Animals (Ministry of Science and Technology of China, 2006) and approved by the animal ethics committees of Jiangnan University (JN. No20180430c0480615).

### 2.2. Preparation

#### 2.2.1. Preparation of the ABL and the HPL

The precursor solution was prepared by dissolving 2 g zein and 2 g EC in 10 mL acetic acid, and the entire solute concentration was maintained at 40% (*w/v*). The solution was stirred intensely at room temperature for 1 h, then, 0.5% PPIX (*w/w*) and 2% Vac (*w/w*) were separately added to obtain the electrospinning solution, which were subsequently stirred for 12 h. The obtained zein/EC/Vac and zein/EC/PPIX electrospinning solutions were, respectively, placed in syringes with a blunt-end metal capillary nozzle with a 1-mm core diameter. The parameters of the electrospinning apparatus were set to a voltage power (18 kV), a constant flow rate at 0.5 mL/h, and a distance of 15 cm between the nozzle and collector. The nanofibers were deposited on the aluminum foil, which covered a circular rotating drum at room temperature.

#### 2.2.2. Preparation of the RFL

*G. xylinum* was inoculated to the fermentation medium (D-mannitol 25 g/L, yeast extract 5 g/L, tryptone 3 g/L) in flakes and shaken gently so that the bacteria were evenly distributed in the culture medium. The bacterial cellulose membrane was formed after static incubation for a week at 30 °C. The inoculated fermentation medium was also added into the petri dishes containing PETN, and then sealed with plastic wrap to culture the BC/PETN membrane. The obtained BC and BC/PETN membrane were purified in 4 wt % NaOH solution to remove any bacteria and culture liquid, followed by rinsing with distilled water and freeze-drying.

#### 2.2.3. Preparation of the MC Membrane

The MC membrane was prepared by using the zein/EC/PPIX nanofibrous membrane as the ABL, the BC/PETN membrane layer as the RFL, and the zein/EC/Vac nanofibrous membrane as the HPL. The BC/PETN membrane was used as a receiver to electrospin the zein/EC/PPIX and zein/EC/Vac solutions onto both sides of the BC/PETN. After hot pressing several times by the roller under the pressure of 1 MPa, the MC membrane was formed.

### 2.3. Characterization

#### 2.3.1. Scanning Electron Microscopy (SEM)

The microstructure and morphology of the samples were examined by an SEM at an accelerating voltage of 5.0 kV. The composite membranes were cut to expose the cross section, which was stuck on the microscope with a conductive adhesive. Before SEM imaging, a layer of gold was applied to the surface of the dried samples to avoid any sample charging effects. The images were collected at 200×, 1000×, or 5000× magnification.

#### 2.3.2. Fourier Transform Infrared Spectroscopy (FTIR)

A Thermo Fisher Scientific FTIR Nicolet iS10 spectrometer was used to record the FTIR spectrum of the samples in the range of 650–4000 cm^−1^, and 16 scans were performed at a resolution of 4 cm^−1^.

#### 2.3.3. Antibacterial Activity Assessment of the ABL

*Staphylococcus aureus* ATCC-6538 and *Pseudomonas aeruginosa* CMCC(B) 10104, obtained from our laboratory, were cultured with Luria–Bertani medium (LB) on a rotary shaker at 37 °C and 200 rpm. When growth of bacteria was monitored by measuring the optical density (OD; 0.6–0.7, 600 nm), we inoculated 0.1 mL of bacterial solution evenly on the surface of the culture medium plates. Samples were prepared by cutting the materials into round-shaped disks with diameters of 10 mm. The zein/EC/PPIX membrane was tested as the treated sample, while zein/EC was set as the control, both of which were placed in bacteria-containing agar plates. After illumination for 30 min, plates were incubated under 37 °C for 24 h in a thermostatic incubator. The formed inhibition zone was recorded and the diameter was measured to calculate the average width.

#### 2.3.4. Mechanical Properties of the RFL

An electronic universal testing machine (KDIII-5) was applied to test the tensile mechanical properties of the BC membranes (dry and wet, respectively) and the BC/PETN composite membranes. The pretension was set as 50 cN, the distance between two clamps that hold the sample was 30 mm, the effective stretching length was 50 mm, and the testing speed was 10 mm/min under the temperature of 25 °C and relative humidity of 65%. Every sample was tested 5 times, and then the breaking strength, elongation at break, and Young’s modulus were calculated.

#### 2.3.5. Hygroscopicity Test of the RFL

The hygroscopicity test was carried out according to the Chinese standard YY/T 0471.1-2004 at room temperature (21 ± 2 °C) and a relative humidity of 60% ± 15%. To determine the swelling behavior of the BC and BC/PETN membranes, samples were prepared to a size of 2 cm × 2 cm, weighed (the weight was set as M_0_), and immersed into simulated body fluids at 37 °C [20]. The swelling rate change of all samples and the different swelling environments were measured at a specific time point until the equilibrium as reached by using the following Formula (1):

Q = (M_1_−M_0_)/M_0_×100% (1)

where Q is the swelling ratio, while M_1_ represents the membrane weights at a specific time. The tests were performed in triplicate.

#### 2.3.6. In Vitro Biocompatibility Assay of the HPL

The zein/EC/Vac nanofibrous membrane was plated at the bottom of the 24-well plates after sterilization. L929 cells were cultured in Dulbecco’s modified Eagle’s medium (DMEM; Gbico) with 10% fetal bovine serum (FBS; Gbico), and 100 U/L penicillin and 100 μg/mL streptomycin (Gibco, Carlsbad, CA, USA). The cells were incubated at 37 °C under an environment with 5% CO_2_. When grown to the logarithmic growth phase, L929 cells were incubated in the 24-well plates with the sterilized zein/EC/Vac nanofibrous membrane by DMEM medium containing 10% FBS. After 24 h of incubation, following with removal of the culture medium with 3–5 times washing in PBS (phosphate buffer saline), PBS with 2.5 % (*w/w*) glutaraldehyde was added at 4 °C for 4 h. Finally, the solution was removed and the membranes were washed with PBS 3 times, then dried at room temperature. The morphology of the L929 cells growing on the surface of the zein/EC/Vac nanofibrous membrane was observed by SEM.

### 2.4. In Vivo Animal Experiments

#### 2.4.1. Establishment of a Skin Wound Model

**Figure 2:**
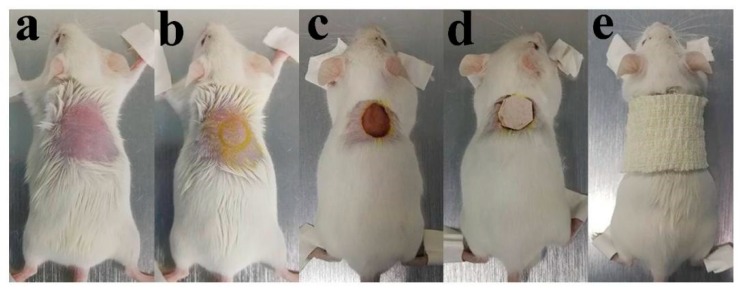
Process of the skin defect preparation: (**a**) fixed and shaved after anesthesia; (**b**) skin disinfection and mark on skin; (**c**) wound modeling; (**d**) dressing on wound; and (**e**) bandaging.

Thirty-six male ICR mice (8 weeks old, 25–30 g) were randomly divided into three groups—sterile gauze group (Control), multilayer composite membrane group (MC), and Nano-Ag dressing group (Nano-Ag), respectively. Mice were fixed, shaved, and sterilized after anesthesia by intraperitoneal injection of 50 mg/kg pentobarbital sodium solution. Sequentially, a round full-thickness skin defect with a diameter of 10 mm was performed on the back of the mice. A digital camera was used to record the shape of the wound, and the diameter of the wound was scaled and compared. Finally, each wound was bandaged with wound dressings from different groups and the mice were fed in different cages. The process of the skin defect preparation is shown in Figure 2.

#### 2.4.2. Wound Observation

Digital photographs of the wounds were recorded by a digital camera and wound size was measured on the day of operation and on various postoperative days (3rd, 7th, and 10th). The wound healing rate was calculated by the difference between the original wound area and the wound area on the 3rd, 7th, and 10th postoperative day as a percentage of the original wound area.

#### 2.4.3. Histological Analysis

Skin samples were collected after 3rd, 7th, and 10th day of treatment with various dressings (4 mice were sacrificed at each time point in each group), immersed in 4% paraformaldehyde, and paraffin-embedded sections were stained with hematoxylin and eosin (H&E; Baso, Taipei, Taiwan). All of the pathological sections were observed under a light microscope (Leica DM4000B, Leica, Wetzlar, Germany).

### 2.5. Statistical Analysis

All data are presented as mean ± SD and analyzed using Student’s *t*-test or one-way ANOVA followed by Duncan’s multiple range test. *p* < 0.05 was considered statistically significant.

## 3. Results and Discussion

### 3.1. Morphological and FTIR Analysis

Zein, a natural polymer material, has previously been broadly prepared by the electrospinning technique to be used as a drug carrier [21,22]. However, it is difficult to maintain the fiber morphology of zein because of its unstable and extremely swellable nature in water, limiting its application as a drug carrier [23]. Ethyl cellulose (EC) has high water stability, better general stability, fiber-forming properties during electrospinning, and good biocompatibility, which makes it a great potential for application in wound dressings [24]. Therefore, we used these two materials to electrospin, loading them with the drugs Vac and PPIX as the ABL and the HPL, respectively, and then the morphology of the different membranes was evaluated.

**Figure 3:**
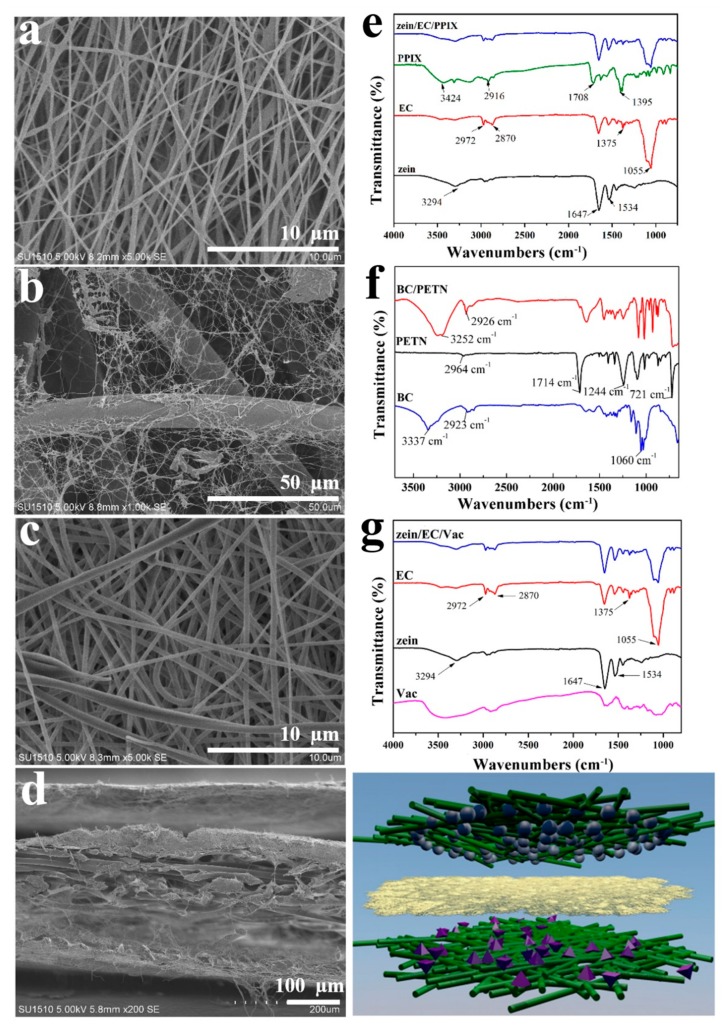
Scanning electronic microscope (SEM) images of the ABL (**a**), the RFL (**b**), the HPL (**c**), and the cross-section of the MC (**d**); FTIR spectra of the ABL (**e**), the RFL (**f**), the HPL (**g**), as well as raw materials.

The nanostructure and three-dimensional network structure of the ABL can be clearly seen in Figure 3a. The nanofibers with an average diameter of 380 ± 92 nm were smooth and continuous without droplets, beads, adhesion, or dissolution. The crystallization and distribution of the nanofibers were uniform and well formed with high spinnability. Figure 3b shows the planar SEM image of the RFL. The nanofibers from BC were intertwined with the fibers of the PETN nonwoven fabric, and part of the BC nanofibers could intersperse between the PETN fibers, forming a three-dimensional network structure. We concluded that the BC fibers produced by the microorganisms firstly attached to the randomly arranged PETN fibers, and then synthesized a large number of BC nanofibers, which filled the pores and surface of the PETN to form a topological network structure [25]. The BC nanofibers were self-assembled and compounded with the nonwovens in the form of a cellulose membrane and a nanofibrous network, in which the BC membrane adhered to the surfaces of the nonwovens and the BC nanofibers interspersed into the voids between the PETN fibers to form a tightly bonded BC/PETN composite membrane [25,26]. An SEM image of HPL is shown in Figure 3c, which demonstrates that the surface of the composite nanofibers was smooth without droplets, beads, adhesion, or dissolution. Zein/EC/Vac fibers with an average diameter of 414 ± 102 nm were irregular, which might be due to the increased surface tension of the mixed solution by Vac, making the jet difficult to differentiate in the electric field. Due to the surface tension of the mixed solution, the composite droplet outside the spinning nozzle was unstable when being drafted, leading to the fiber diameter being uneven and to an increasing fiber discrete degree. In addition, the drug was dissolved in the solvent and existed in the form of ions, which caused charge accumulation on the surface of the jet and divided the jet into smaller jets under relatively high electric field intensity [27]. All of the above situations would result in uneven fibers. The SEM image of the cross-section of the multilayer composite membranes shows a three-layer nanofibrous structure with an average thickness of 143 ± 10.35 μm (Figure 3d).

Figure 3e displays the FTIR spectrum of the ABL nanofibers. The broad peak at 3294 cm^−1^ in the spectra of zein was due to the stretching vibration of N=H and O=H of the protein chain, and the characteristic bands observed at 1647 cm^−1^, 1534 cm^−1^, and 1230 cm^−1^ corresponded to C=O stretching (amide I) and N=H bending vibration (amide II) [28]. The spectra of EC illustrate that the peaks at 2972–2870 cm^−1^, 1375 cm^−1^, and 1055 cm^−1^ represent the stretching vibration of –CH_3_, the bending vibration of –CH_3_, and –C–O–C stretching in the cyclic ether [29,30]. From the FTIR spectra of PPIX, three obvious characteristic peaks of stretching vibration at 3424 cm^−1^ (N–H), 2916 cm^−1^ (C–H), and 1708 cm^−1^ (C=O) could be observed. The absorption band at 1200–1500 cm^−1^ was the skeleton vibration of the porphyrin ring, which could be attributed to the symmetric and asymmetric absorption peaks of the C–H, C=C, and C=N bonds in the pyrrole ring [31]. The spectrum of the zein/EC/PPIX composite nanofibers had the characteristic absorption peaks of PPIX, zein, and EC without new absorption peaks, indicating that the components in the composite nanofibers were only physically mixed rather than chemically bonded. Figure 3f shows the FTIR spectra of the RFL. The characteristic absorption peaks of PETN at 2964 cm^−1^, 1714 cm^−1^, 1244 cm^−1^, and 721 cm^−1^ were assigned to the stretching vibration absorption peaks of –CH_2_, –C=O, and –C–O–C in the polyethylene terephthalate macromolecular structure and the bending vibration of the –CH bond on the benzene ring, respectively [25]. Due to the large number of hydroxyl groups on the surface of the BC membrane, it presented a strong characteristic absorption band at 3337 cm^−1^ [32]. From Figure 3f, a red-shifted hydroxyl absorption peak was observed at 3252 cm^−1^ after forming a composite with PETN, while the –CH stretching vibration peak around 2926 cm^−1^ was enhanced by the –CH bond in BC and PETN, implying that a good physical composite was formed between BC and PETN. From Figure 3g, the FTIR spectra of the Vac show three characteristic peaks at 3450 cm^−1^, 1654 cm^−1^, and 1450 cm^−1^, which were associated with the stretching vibrations of O–H, C=O, and C=C, respectively [33]. The interactions between polymer and drug molecules such as the hydrogen bond, electrostatic adsorption, and hydrophobic effect would increase compatibility while preparing the composite nanocellulose membrane [34]. Zein and Vac possessed free hydroxyl and carboxyl groups, generating a hydrogen bond interaction in the composite nanofiber [35]. It was noted that no new absorption peak appeared in the FTIR spectrum of the zein/EC/Vac nanofibrous membrane, indicating that only physical mixing existed among the components in the drug-loaded nanofibers. It was testified that the raw materials could maintain their original activity and properties without chemical reaction.

### 3.2. Antibacterial Activity Assessment of the ABL

**Figure 4:**
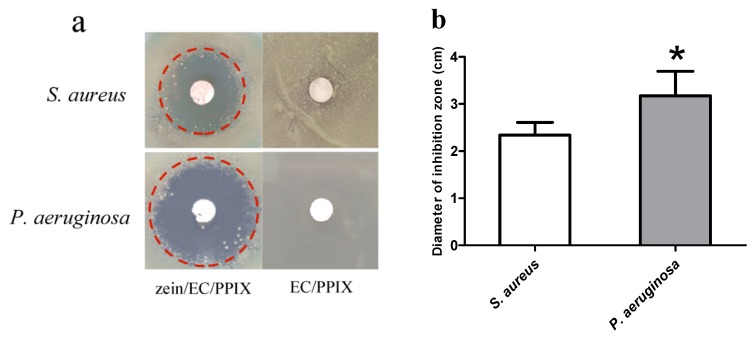
(**a**) The images of inhibition zones obtained by incubating *S. aureus* or *P. aeruginosa* with the zein/EC/PPIX nanofibrous membrane (ABL) and the EC/PPIX nanofibrous membrane. (**b**) Diameter statistics of the inhibition zone. * represents a statistically significant difference from the *S. aureus* group (*p* < 0.05).

Figure 4a shows pictures of the inhibition zones obtained by incubating *S. aureus* or *P. aeruginosa* with the ABL membrane. The zein/EC nanofiber membranes displayed no inhibition zone, suggesting that it failed to exert an inhibition effect on *S. aureus* or *P. aeruginosa*, while the ABL membrane displayed a significant inhibition zone after the excitation by illumination. Protoporphyrin IX (PPIX), as one of the common photosensitizers, was shown to exert a significant antibacterial effect on *S. aureus* and *E. coli*. We speculated that the oxygen-free radicals generated by composite membranes loaded with PPIX can cut off the major chemical bonds, such as O–P, C–H, O–H, and N–H bonds of bacteria, then bacteria are decomposed and the ABL exerts an effect of sterilization [36]. On the other hand, the PPIX could form singlet oxygen when being excited by illumination, which would destroy the cell membrane structure of the bacteria, reducing the vitality of the cell and inhibiting cell growth [31].

Furthermore, we also found that the inhibition zone of the ABL membrane in the *P. aeruginosa* group was significantly bigger than that in the *S. aureus* group (*p* < 0.05, Figure 4b). *P. aeruginosa*, also named green pus bacillus and belonging to the category of Gram-negative bacteria, is one of the major causes of hospital infections and can lead to postoperative wound infection [37]. *S. aureus*, as the representative bacterium of Gram-positive bacteria, can cause many serious infections and is the most common pathogen in human suppurative infection [19]. Compared to Gram-negative bacteria, Gram-positive bacteria contain peptide polysaccharide with a dense reticular structure in the thick cell wall [38]. The present study indicates that the PPIX-loaded zein/EC nanofibrous membrane possessed better antibacterial effects against Gram-negative bacteria, which could be relevant to the relatively thick cell wall of Gram-positive bacteria. The exact mechanism still needs further study.

### 3.3. Mechanical Properties of the RFL

BC is considered a very promising wound dressing [39]. However, wet BC is difficult to reprocess and dry BC membranes possess lower mechanical properties because of the porous matrices, showing some restricted application in wound dressings [40]. To achieve an ideal material, nonwoven composites with BC have shown more adequate mechanical properties in the literature [41].

As can be seen from Table 1, the BC/PETN membrane had a significantly increased elongation at the break in comparison with the dried BC membrane, which was similar to that of the wet BC membrane, suggesting that the BC/PETN membrane had good malleability. This was mainly due to the high elongation at the break of PETN itself [42]. Additionally, BC nanofibers with a network structure were filled in between the PETN fibers, and the close entanglement of the fibers greatly improved the mechanical properties of the composite membranes [43].

The dried BC membrane possessed a relatively higher Young modulus and larger rigidity, while the BC/PETN membrane had a decreased Young modulus in contrast to the dried BC membrane, still retaining certain strength. Considering the absorption performance, reprocessing, and easy preservation, the dried BC membrane was set as a layer of the multilayer composite structure for application research on wound dressings. However, the dried BC membrane was easy to break and difficult to preserve or reprocess. Non-woven fabric PETN was added for structural modification during BC membrane formation to modify the mechanical properties of the dried BC membrane with the mechanical effects generated from the entanglement of BC on PETN fibers and the original hydrogen bond interaction among the BC fiber.

The results revealed that the elongation at the break was largely improved, the flexibility was also increased, and malleability and strength were obtained when the BC membrane was combined with a non-woven fabric such as PETN to form composite materials. It successfully overcame the shortcomings of the dried BC membrane, being light, thin, crispy, and hard to spread. Meanwhile, the BC/PETN membrane was dry, flat, soft, resilient, and easily reprocessed, which laid the foundation for RFL application in wound dressing.

### 3.4. Hygroscopicity Test of the RFL

Modern wound-healing theories confirm that a moist healing environment can provide suitable growth conditions for cells in the damaged skin [44]. Meanwhile, an eligible wound dressing not only provides a moist and suitable microenvironment, but also reduces local pain and secondary injury caused by the removal of the dressing for wound healing [45]. However, the local wound skin becomes immersed and weakens the defense barrier against bacterial invasion when wound exudate increases. Therefore, timely drainage of wound exudate is needed to promote the healing of the wound. The ability of the wound dressing to absorb or expel wound secretions can be determined by a hygroscopicity test of the wound dressing [46].

**Figure 5:**
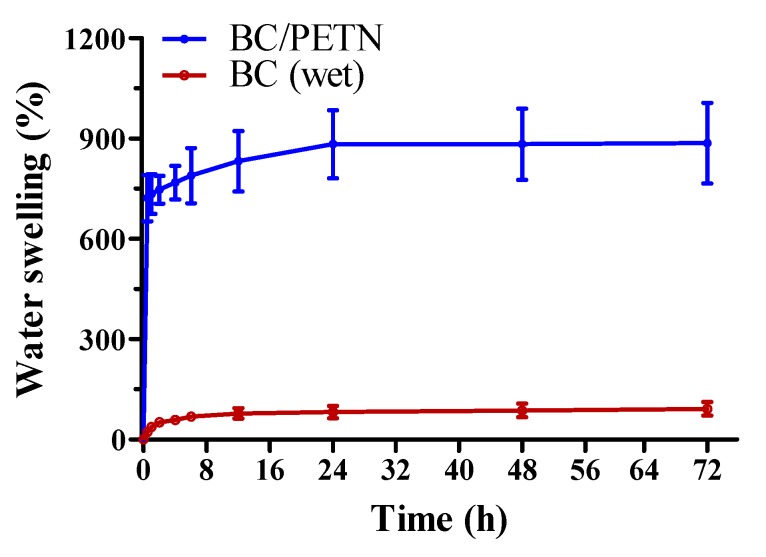
Water swelling ratio of the BC/PETN and BC (wet) membranes.

In this experiment, the BC/PETN and wet BC membranes were put into SBF solution at 37 °C, respectively, to test their hygroscopicities within 72 h (Figure 5). Both of the samples had a fast swelling speed within 0.5–1 h, which then gradually decreased. The BC/PETN membrane reached equilibrium at 24 h with a swelling ratio of 883%, indicating a good swellability. Although the wet BC membrane had hygroscopic capacity with a water swelling ratio of 82% at 48 h, its hygroscopic ability was much lower than that of BC/PETN, which was related to the fact that the wet BC membrane contained a large number of water molecules. The good hygroscopicity of the BC/PETN composite membrane was attributed to the rich internal porous structure of BC and a three-dimensional network, resulting in a large specific surface area and a large space for adsorption of water molecules. In addition, the polyhydroxyl structure of BC and the upper and lower BC surface of the composite membrane also contributed to strong hydrophilicity and water swellability [47]. Therefore, the BC/PETN composite membrane, as one of the basic structures of a wound dressing, was conducive to the absorption of wound exudate and could meet the requirements as a wound dressing.

### 3.5. In Vitro Biocompatibility Evaluation of the HPL

**Figure 6:**
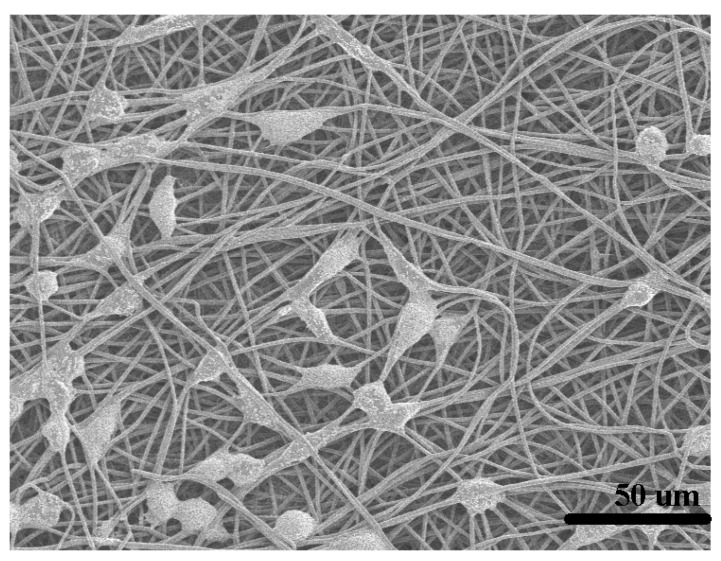
SEM image of L929 cells cultured on the zein/EC/Vac membrane.

Evaluation of the cell growth on the zein/EC/Vac nanofibrous membrane was observed through a cell direct contact test. As shown in Figure 6, L929 cells, growing on the surface of the nanofibrous membrane, exhibited favorable morphology in the shape of flat polygons or long spindles, and the cells extended well with a relatively large number of cells. This implies that the zein/EC/Vac nanofibrous membrane would be an ideal material for cell growth, with good biocompatibility.

Vaccarin (Vac), an extract of Vaccariae semen, showed properties of promoting specific proliferation and migration of vascular endothelial cells, contributing to neovascularization [48]. Vac loaded on the BC membranes as a wound dressing exhibited a beneficial effect due to Vac being released to promote endothelial tissue proliferation [33]. Due to the good proangiogenic effect of Vac [49], the zein/EC membrane loaded with Vac as the HPL has a good potential for application in the field of wound dressings.

### 3.6. In Vivo Animal Experiment

#### 3.6.1. Wound Observation

**Figure 7:**
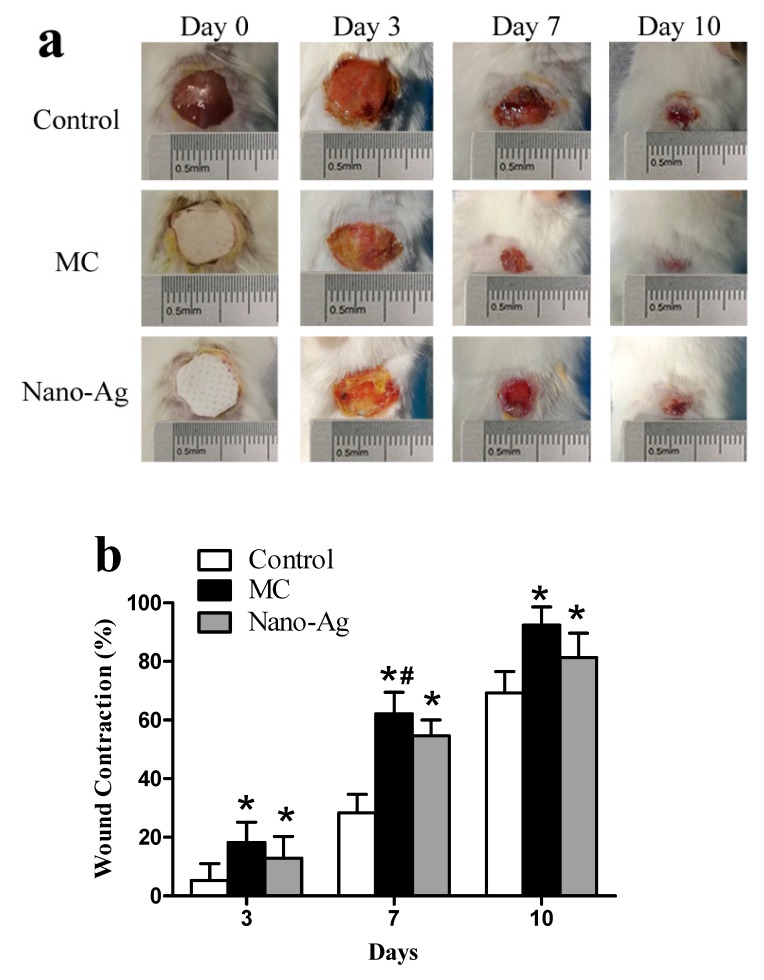
(**a**) Photographic images on days 0, 3, 7, and 10 of wounds treated with sterile gauze (Control group), multilayer composite membranes (MC group), and Nano-Ag dressing (Nano-Ag group). (**b**) Analysis of the wound healing rate in each group on days 3, 7, and 10. * represents a statistically significant difference from the Control group (*p* < 0.05). # represents a statistically significant difference from the Nano-Ag group (*p* < 0.05).

We conducted an experiment on skin wounds using different dressings (Figure 7a). The Control group showed more severe inflammation, with redness and swelling appearing around the wound on day 3. Both the multi-layer composite wound dressing group (MC group) and the Nano-Ag wound dressing group (Nano-Ag group) promoted a small amount of new epithelial tissue proliferation, while the Nano-Ag group showed some exudate on the wound surface. On day 7, the average wound healing rates of the three groups were 28.35% (Con), 62.15% (MC), and 54.63% (Nano-Ag), respectively. As demonstrated in Figure 7b, the wound contraction in the MC group was remarkably higher than that in the Control and Nano-Ag groups. On day 10, the wound contraction of the MC and Nano-Ag groups reached 92.4% and 81.3% respectively, while the wound of the control group was still obvious.

We also found that the Nano-Ag dressing adhered to desiccated wound surfaces when the dressing was not removed for a long time, which induced trauma on removal. The BC layer in the MC membrane could maintain the wet healing environment of the wound due to its strong hygroscopicity. Before the dressing was removed, we firstly moistened the dressing with saline to make it reach full absorption and keep it moist, which could effectively avoid the occurrence of reinjury.

#### 3.6.2. Histological Analysis

**Figure 8:**
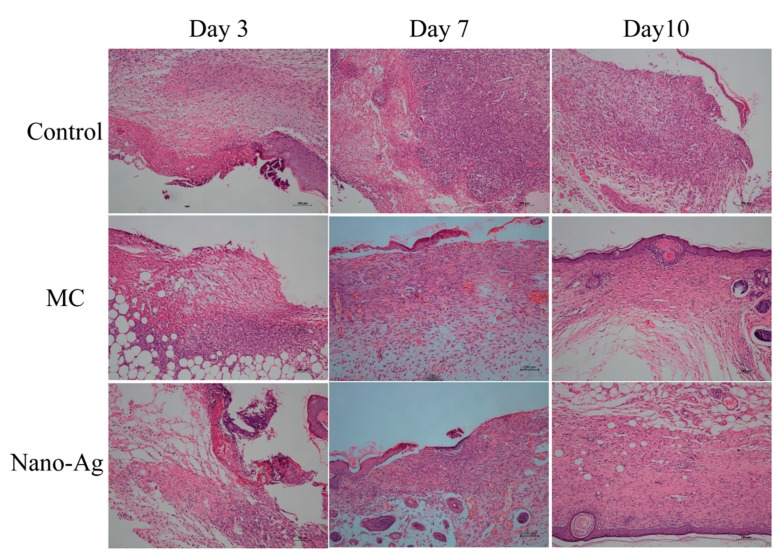
Histological images on days 3, 7, and 10 of the wounds treated with sterile gauze (Control group), multilayer composite membranes (MC group), and Nano-Ag dressing (Nano-Ag group).

Wound healing is a complex biological process involving multiple cells and tissues, including tissue regeneration, epithelialization, and granulation tissue formation [50]. The pathological changes of skin wound tissues at different time points can be observed by H&E staining. As shown in Figure 8, on day 3, a great amount of deeply colored inflammatory cells infiltrating and accumulating on the wound surface could be observed in the Control group. However, the MC group exhibited the least inflammatory cells and a small amount of neovascularization (oval, mostly on the wound surface), while some inflammatory cell infiltration and a small amount of neovascularization were observed in the Nano-Ag group.

On day 7, there were still a large number of inflammatory cells infiltrating the Control group without regular skin structure and no epidermal structure. The MC group showed less inflammatory cells and more new neovascularization and fibroblasts, and thus had basically completed epithelialization. In the Nano-Ag group, part of the epidermis and the basic epidermal structure with a few fibroblasts and abundant new neovascularization could be observed.

On day 10, the epidermal structure in the Control group was not yet completely formed. The MC and Nano-Ag groups had produced a large amount of collagen fibers on the surface of the wound, forming the structure of the epidermis. Each layer was regular and orderly on the wound surface, similar to normal skin [51]. Meanwhile, the granulation tissue had formed and grown into the wound, which was filled with granulation tissue and had begun fibrosis in the MC and Nano-Ag groups.

## 4. Conclusions

In conclusion, an MC membrane—including an ABL (zein/EC/PPIX), an HPL (zein/EC/Vac), and an RFL (BC/PETN)—used as wound dressing material was successfully prepared. Each layer was fully characterized and analyzed, the results of which confirmed that the ABL could effectively inhibit *Staphylococcus aureus* and *Pseudomonas aeruginosa*, and the RFL prepared by the in situ biological composite method possessed a desirable nanonetwork structure and exhibited good flexibility, mechanical properties, and good hygroscopicity. In addition, the HPL favored biocompatibility in vitro. The animal experiment showed that the wound healing rate of mice treated in the MC group reached 92.4% on day 10, which was significantly higher than that of the Control group. Histological examinations demonstrated that active fibroblasts and epithelialization could be observed, and granulation tissue was filled in the wounds to complete the process of tissue remodeling treated by MC and commercial Nano-Ag dressings. Given the general properties of the MC membrane and its satisfactory performance in the mouse wound model, the MC membrane demonstrates a promising future in the design and production of next-generation wound dressing materials. 

## Figures and Tables

**Table 1:** The mechanical properties of the BC and BC/PETN membranes.

Sample	Breaking Strength (MPa)	Elongation at Break (%)	Young Modulus (MPa)
Dried BC	7.85 ± 2.07	6.33 ± 1.64	93.6 ± 16.4
Wet BC	3.39 ± 0.65*	34.39 ± 5.32*	19.4 ± 3.27*
Dried BC/PETN	3.40 ± 0.34*	28.88 ± 2.03*	11.77 ± 1.90*

* represents a statistically significant difference from the dried BC group (*p* < 0.05).
